# Application of Molecular Hydrogen as a Novel Antioxidant in Sports Science

**DOI:** 10.1155/2020/2328768

**Published:** 2020-01-14

**Authors:** Takuji Kawamura, Kazuhiko Higashida, Isao Muraoka

**Affiliations:** ^1^Faculty of Sport Sciences, Waseda University, 2-579-15 Mikajima, Tokorozawa, Saitama 359-1192, Japan; ^2^Department of Food Science and Nutrition, The University of Shiga Prefecture, 2500, Hassaka-cho, Hikone, Shiga 522-8533, Japan

## Abstract

Molecular hydrogen (H_2_) is a colorless, tasteless, odorless, and minimal molecule with high flammability. Although H_2_ has been thought to be an inert gas in living bodies for many years, an animal study reported that inhalation of H_2_ gas decreased oxidative stress and suppressed brain injury caused by ischemia and reperfusion injury due to its antioxidant action. Since then, the antioxidant action of H_2_ has attracted considerable attention and many studies have reported on its benefits. Most studies have reported the effects of H_2_ on diseases such as cancer, diabetes, cerebral infarction, and Alzheimer's disease. However, little is known regarding its effects on healthy subjects and exercise. Thus far, including our study, only 6 studies have explored the effect of H_2_ on exercise. H_2_ is the smallest molecule and therefore can easily penetrate the cellular membrane and rapidly diffuse into organelles. H_2_ is thought to be able to selectively reduce hydroxyl radicals and peroxynitrite and does not affect physiologically reactive species. H_2_ can be supplied to the body through multiple routes of administration, such as oral intake of H_2_ water and H_2_ bathing. Therefore, H_2_ may be a potential alternative strategy for conventional exogenous antioxidant interventions in sports science. The purpose of this review is to provide evidence regarding the effects of H_2_ intake on changes in physiological and biochemical parameters, centering on exercise-induced oxidative stress, for each intake method. Furthermore, this review highlights possible future directions in this area of research.

## 1. Introduction

Molecular hydrogen (H_2_) is a colorless, tasteless, odorless, and minimal molecule with high flammability [1]. Most mammals, including humans, do not synthesize hydrogenase, which is a catalyst for the activation of H_2_ [2], and therefore, H_2_ has long been considered to be an inert gas in mammalian cells. However, in 2007, a pioneer study reported that H_2_ can selectively reduce hydroxyl radicals (^·^OH) and peroxynitrite (ONOO^−^) in cultured cells but not other reactive species, such as superoxide (O_2_^-·^), hydrogen peroxide (H_2_O_2_), and nitric oxide (NO^·^) [3]. The study also demonstrated that inhalation of H_2_ gas markedly decreased oxidative stress and suppressed brain injury caused by ischemia and reperfusion injury in rats [3]. Since the study was carried out, the amount of research on the antioxidant and therapeutic effects of H_2_ has increased rapidly. Moreover, studies have suggested that H_2_ can prevent the progression of various diseases [1, 4–8]. As such, research regarding the medical applications of H_2_ is steadily progressing, with several clinical studies already started [1, 4–8].

In sports science, there is limited research regarding the antioxidant effect of H_2_ on exercise-induced oxidative stress. Unlike conventional antioxidants, H_2_ is a gas molecule and as such it is believed to have several advantages for application in sports science [4, 6, 9] (Table 1). Firstly, H_2_ is the smallest molecule and thus can penetrate the cellular membrane and rapidly diffuse into organelles (e.g., mitochondria). Secondly, H_2_ is thought to have no effect on physiologically reactive species (e.g., H_2_O_2_), as it can selectively reduce ^·^OH and ONOO^−^. Finally, H_2_ can be supplied to the body through multiple routes of administration, such as oral intake of H_2_ water, H_2_ bathing, intravenous infusion of H_2_-saline, and inhalation of H_2_ gas. In addition to these advantages, H_2_ can be used with minimal side effects as it is excreted by exhaling. Despite several disadvantages (Table 1), the aforementioned advantages of H_2_ use are expected to lead to an increase in research regarding its application in sports science.

The purpose of this review is to provide evidence regarding the effects of H_2_ intake on changes in physiological and biochemical parameters, centering on exercise-induced oxidative stress, as well as illustrate the mechanisms underlying the biological actions of H_2_. More specifically, this review describes findings from previous studies regarding the effects of each method of H_2_ administration. Moreover, we also summarize possible future directions for this area of research.

## 2. Biological Actions of Molecular Hydrogen

Although the antioxidative action of H_2_ was suggested in the study by Dole et al. [10] in 1975, its biological action has been overlooked for many years. Later, in 2007, it was reported that H_2_ selectively removes ^·^OH and ONOO^−^, which are strong oxidants, *in vitro* and that H_2_ suppresses oxidative stress after ischemia and reperfusion injury *in vivo* [3]. Since then, H_2_ has attracted widespread interest as a novel antioxidant and numerous previous studies have reported on the effectiveness of H_2_ for various diseases and disease models associated with oxidative stress [1, 4–8]. However, the direct removal of ^·^OH and ONOO^−^ alone cannot fully explain the beneficial effects exerted by H_2_ in these diseases. Therefore, the indirect effects of H_2_ on the regulation of intracellular signaling pathways and gene expression have been investigated [1, 4–8]. Specifically, it has been shown that H_2_ activates Nrf2 (nuclear factor-erythroid-derived 2-like-2) under oxidative stress conditions to increase the gene expression of antioxidant enzymes such as superoxide dismutase (SOD) and catalase [1, 4–8]. H_2_ has also been shown to downregulate the transcription factor NF-*κ*B and inflammatory cytokines (e.g., interleukin- (IL-) 1*β*, IL-6, and tumor necrosis factor (TNF-*α*)) in oxidative stress-induced inflammation [1, 4–8]. Moreover, recent studies have suggested that H_2_ suppresses lipid peroxidation associated with free radical chain reactions [11]

**Figure 1:**
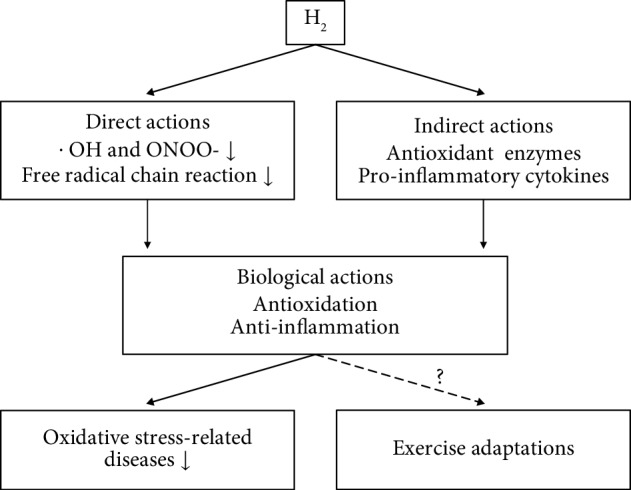
Possible simplified biological actions of molecular hydrogen: focusing on antioxidant and anti-inflammatory actions.

Taken together, the antioxidant action of H_2_ is considered to be not only direct, by selective removal of reactive species [3] and suppression of free radical chain reactions for lipid peroxidation [11], but also indirect, by inducing the expression of antioxidant enzymes. Furthermore, considering that H_2_ downregulates the expression of inflammatory cytokines [1, 4–8], this may also suppress infiltration of phagocytes into the inflammatory site and subsequent release of reactive species. Possible biological actions of H_2_ are shown in Figure 1.

## 3. Exercise-Induced Oxidative Stress

Exercise is one of the physiological stimuli that promote the generation of reactive species in the living bodies. The generation of reactive species by exercise depends on exercise intensity, duration, and modality [12, 13]. The living body is equipped with an enzymatic or nonenzymatic antioxidant defense system. However, oxidative stress occurs when the levels of reactive species surpass the antioxidant capacity of the organism [14]. Exercise-induced oxidative stress has been shown to result in transient declines in physical functions through muscle fatigue, muscle damage and inflammation, and delayed-onset muscle soreness (DOMS) [12, 13, 15]. Moreover, there are many previous studies which have verified the effectiveness of taking exogenous antioxidants [12, 13, 15].

On the other hand, it should be mentioned that long-term excessive intake of exogenous antioxidants inhibits redox-sensitive signaling pathways and interferes with physiological adaptations to exercise training, such as mitochondrial biogenesis, cardiac and skeletal muscle hypertrophy, and improvement of insulin sensitivity [13, 16]. The results of previous studies regarding exercise redox biology indicate that the generation of excess levels of reactive species has a negative effect, while the generation of low-to-moderate levels of reactive species has a positive effect on the living body. The dependence of physiological responses or adaptations on the level of reactive species is called exercise hormesis and may be an important criterion for the optimization of the effects of exogenous antioxidants [17].

## 4. Research on the Application of H_2_ in Sports Science

### 4.1. H_2_ Intake Methods

Six studies (Table 2) have been carried out on the effectiveness of H_2_ in sports science, involving 4 intake methods. Specifically, there are 2 studies on the oral intake of H_2_ water, 2 studies on H_2_ bathing, 1 study on intravenous infusion of H_2_-saline, and 1 study on the inhalation of H_2_ gas. In this section, we introduce previous reports that have investigated the effects of each method of H_2_ administration on changes in physiological and biochemical parameters, centering on exercise-induced oxidative stress and inflammation.

### 4.2. Oral Intake of H_2_ Water

Intake of H_2_ water is one of the most practical and safe intake methods for daily life and in the sports field. H_2_ can be dissolved in water up to concentration of 0.8 mM (1.6 mg/L) under atmospheric pressure at room temperature [4]. However, in order to avoid a decrease in H_2_ concentration, it must be stored in an aluminum container.

Aoki et al. initially reported that oral intake of H_2_ water has no effect on blood reduction/oxidation (redox) biomarkers such as diacron reactive oxygen metabolites (d-ROMs) and biological antioxidant potential (BAP) but suppresses the elevation of blood lactate concentrations and reduces peak torque during exercise in trained young men [18]. Our research group investigated the effects of oral intake of H_2_ water on exercise-induced oxidative stress and its related indicators using an animal model [19]. Similar to the results of Aoki et al. [18], our findings indicated that a 2-week intake of H_2_ water did not affect redox homeostases, such as thiobarbituric acid reactive substances (TBARS), protein carbonyl (PC), and total antioxidant capacity (TAC), in both plasma and skeletal muscle during exhaustive running in fasting rats [19]. In addition, we also demonstrated that H_2_ water intake did not affect blood energy substrates, muscle glycogen content, and performance level, while it slightly suppressed liver glycogen utilization during exercise. In contrast to these results, our latest data showed that a 2-week intake of H_2_ water increases plasma lactate and free fatty acid concentrations, as well as liver glycogen utilization, during constant exercise at low intensity in fed rats (unpublished data).

Taken together, there is no report regarding the effectiveness of H_2_ water intake against exercise-induced oxidative stress and inflammation in humans and animals [18, 19]. On the other hand, there are fragmented reports on the possible effects of H_2_ intake on glucose metabolism [18], liver glycogen utilization [19], and performance levels [18] during exercise.

### 4.3. H_2_ Bathing

Aside from the oral intake of H_2_ water, H_2_ bathing is another method with high applicability in sports. For this method, an H_2_-producing agent is generally used (e.g., MgH_2_). The H_2_-producing agent can be stably stored for long periods and can be used safely and easily. The generated H_2_ is delivered into the body orally and transcutaneously, and it is considered that H_2_ reaches the whole body only 10 min after the H_2_ bath, based on the concentration of H_2_ in the breath [20].

Our research group investigated the influences of weekly H_2_ bathing on exercise-induced oxidative stress and inflammatory responses, as well as muscle damage and DOMS after downhill running [21]. Our findings showed that weekly H_2_ bathing had no influence on redox homeostasis (i.e., TBARS, d-ROMs, and BAP), inflammatory responses (i.e., IL-6, IL-17a, and myeloperoxidase (MPO)), and the degree of muscle damage markers (i.e., creatine kinase (CK) and myoglobin (Mb)) in the blood. However, weekly H_2_ bathing alleviated DOMS as evaluated by the visual analogue scale 24 and 48 h after downhill running. We also investigated the effects of weekly H_2_ bathing on neutrophil dynamics and function, which play an important role in secondary oxidative stress after eccentric exercise [21]. Our results showed that H_2_ bathing after downhill running did not influence the peripheral neutrophil count or its functions, such as migration activity and reactive oxygen species (ROS) productivity, as evaluated by luminol-dependent chemiluminescence (LmCL).

As described above, there is no conclusive evidence regarding the effectiveness of H_2_ bathing against muscle damage, secondary oxidative stress, and inflammation after eccentric exercise [21, 22]. Moreover, although the associated mechanism of action has not been elucidated, our results have shown that H_2_ may alleviate DOMS after eccentric exercise [21].

### 4.4. Intravenous Infusion of H_2_-Saline

Intravenous infusion of H_2_ saline is a method that can rapidly supply a large amount of H_2_ into the living body. However, this method may be difficult to use in the sports field due to its invasiveness.

Yamazaki et al. investigated the effects of an intravenous infusion of H_2_-saline on blood redox and metabolic/injury biomarkers in thoroughbred horses after a high-intensity simulation race [23]. Their results showed that an intravenous infusion of H_2_-saline decreased serum 8-hydroxydeoxyguanosine (8-OHdG), which reflects the development of DNA damage. However, there was no significant difference in the level of other redox (i.e., d-ROMs and BAP) and metabolic/injury biomarkers (e.g., lactate, uric acid, and CK) in the blood after the simulation race. Therefore, there is limited evidence regarding the efficacy of H_2_-saline infusion.

### 4.5. Inhalation of H_2_ Gas

From the viewpoint of experts in sports science, the inhalation of H_2_ gas is not a versatile method of H_2_ administration. However, this method can quickly supply a large amount of H_2_ to the living body. H_2_ gas can easily be inhaled through a ventilator circuit, face mask, or nasal cannula, and there is no risk of explosion when the concentration in the air is below 4% [20].

Nogueira et al. reported that inhalation of 2% H_2_ gas while treadmill running suppresses TBARS levels and inflammatory biomarkers, such as TNF-*α* and IL-6, in rat plasma immediately or 3 h after exercise [24]. In addition, plasma SOD activity 3 hours after exercise was enhanced by H_2_ intake. This previous study also showed that the phosphorylation of skeletal muscle cAMP-responsive element binding (CREB) protein, which is involved in increasing oxidative metabolism and mitochondrial biogenesis, is attenuated by the inhalation of H_2_ gas at 3 h after exercise. Therefore, the inhalation of H_2_ gas simultaneously with exercise is effective in suppressing exercise-induced oxidative stress and inflammation but may inhibit the adaptation of skeletal muscle by exercise training.

### 4.6. Summary of the Results of Previous Studies

Although the effectiveness of oral intake of H_2_ water [18, 19] and H_2_ bathing [21, 22] has not been demonstrated, intravenous infusion of H_2_-saline [23] and inhalation of H_2_ gas [24] have been reported to suppress exercise-induced oxidative stress and/or inflammation. However, the antioxidant and anti-inflammatory effects of H_2_ intake have been observed only in animals [23, 24], and these effects have not been confirmed in humans regardless of the administration route [18, 19, 21, 22]. Regarding the timing of H_2_ administration, it seems to be most effective before [23] or simultaneously with exercise [24].

Other than its antioxidant and anti-inflammatory effects, some studies have partly shown that H_2_ intake exerts some effects such as improvement of exercise performance [18], changes in glucose metabolism [18] and liver glycogen utilization [19], and alleviation of DOMS [21]. However, it should be mentioned that, like other antioxidants, H_2_ intake may partially inhibit physiological adaptations induced by exercise training [24].

## 5. Future Directions

Research on H_2_ for applications in sports science is in its incipient stages. Given the advantages of H_2_ (Table 1), it may be worth investigating the effects of H_2_ intake on physiological and biochemical responses, especially exercise-induced oxidative stress and inflammation.

Firstly, as a future effort, it will be necessary to establish an optimal H_2_ intake protocol based on the dynamics of H_2_ in the body. In particular, among the methods of H_2_ intake, oral intake of H_2_ water and H_2_ bathing seem to be the most practical intake methods that can be used even in the general sports field. To date, few papers have reported changes in H_2_ concentration in the living body after H_2_ water intake and H_2_ bathing [20, 25]. This suggests that either method of administration peaks *in vivo* between 5 and 10 minutes and then returns to baseline values by 60 minutes [20, 25]. Therefore, in order to obtain an acute H_2_ effect, administration immediately before or during exercise may be more effective. As such, when verifying the acute effect, oral intake of H_2_ water may be more practical than H_2_ bathing. Since the effects of H_2_ are influenced by various factors, such as intake method, timing, concentration, dose, and frequency, it is of great importance to steadily accumulate evidence regarding the effects of H_2_ on reducing exercise-induced oxidative stress and inflammation.

Secondly, in addition to the H_2_ intake protocol, it is also necessary to conduct a study that takes into account the individual redox properties of the subjects. In recent years, the importance of personalized antioxidant strategies has been proposed [26]. Specifically, beneficial effects such as reducing exercise-induced oxidative stress and improving exercise performance due to ingestion of exogenous antioxidants are only seen in subjects with insufficient resting antioxidant levels, but these beneficial effects have not been observed in subjects with appropriate antioxidant levels at rest [27, 28]. Therefore, it is required to examine the effectiveness of H_2_ intake on exercise-induced oxidative stress and related indicators after screening the resting antioxidant status of the subjects.

Thirdly, the effects of long-term H_2_ intake on exercise adaptations must be clarified. As mentioned above, long-term and excessive antioxidant (e.g., vitamin C and vitamin E) intake has been shown to inhibit redox-sensitive signaling pathways and interfere with physiological adaptations to exercise training [13, 16]. Although several studies have presented counterevidence [29, 30], long-term and excessive exogenous antioxidant intake should be avoided during exercise training. Unlike conventional antioxidants, H_2_ has been postulated to selectively reduce ^·^OH and ONOO^−^ and not affect physiologically reactive species. However, previous studies have shown [24] that the phosphorylation of skeletal muscle CREB is attenuated after exercise by the inhalation of H_2_ gas. This suggests that H_2_ may interfere with the beneficial effects of exercise training by inhibiting cellular signaling pathways during acute exercise stimulus. Therefore, it is important to clarify the effect of chronic H_2_ intake on physiological adaptations induced by long-term exercise training (Figure 1).

Fourthly, it will be necessary to unravel the alternative mechanism underlying the antioxidant action of H_2_. Previous studies have shown that H_2_ intake is effective in improving exercise performance [18], changing the glucose metabolism and liver glycogen utilization [18, 19], and mitigating DOMS [21] without affecting redox biomarkers. On the other hand, several studies have investigated the effects of H_2_ on the buffer capacity of the blood, exhaled gas parameters, and exercise performance without measuring redox biomarkers. As such, there is only fragmented evidence regarding the positive effects of H_2_ intake [31–38]. Notably, many findings regarding the medical efficacy of H_2_ could not be explained solely by selective removal of reactive species [3] and suppression of free radical chain reactions for lipid peroxidation [11]. However, research on the action mechanism of H_2_ has just started, and further research development is expected in the future.

## 6. Conclusions

Here, we briefly summarized the current findings regarding the effect of H_2_ intake on changes in physiological and biochemical parameters, centering on exercise-induced oxidative stress. However, presently, there are few studies aimed at applying H_2_ in sports science and its effectiveness and long-term effects have not been fully demonstrated. Therefore, it is premature to conclude its usefulness. Since H_2_ is a gas molecule and has several advantages, it is worthwhile to continue research towards the application of H_2_ in sports science.

## Figures and Tables

**Table 1:** Advantages and disadvantages of molecular hydrogen.

Advantages
Easily penetrates the cellular membrane and rapidly diffuses into organelles
Selectively reduces ^·^OH and ONOO^−^ and does not affect physiological reactive species
Can be supplied to the body through multiple routes of administration
Can be used with minimal side effects as it is excreted by exhaling
Disadvantages
Dwells in the body for a short time
The optimal intake protocol has not been established
The effects of long-term intake are unknown
Small number of studies

**Table 2:** Effects of molecular hydrogen on exercise-induced oxidative stress, inflammation, and other indicators.

Method	Study	Subjects	Intake protocol	Exercise	Markers	Effects
H_2_ water	Aoki et al. [18]	10 T	500 mL × 3 before Ex	Cycling (75% VO_2_ max) Isokinetic knee extension	d-ROMs, BAP	→
					CK	→
					Lactate	↓
					Peak torque	↑
	Kawamura et al. [19]	32 rats	*Ad libitum* 14 d before Ex	Running (exhaustion)	TBARS	→
					PC	→
					TAC	→
					Lactate	→
					Glucose, FFA, TG	→
					Muscle glycogen	→
					Liver glycogen	↑

H_2_ bathing	Kawamura et al. [21]	9 UT	20 min Immediately and 1–6 d after Ex	Downhill running (56% VO_2_ max)	DOMS	↓
					CK, Mb	→
					Lactate	→
					TBARS	→
					d-ROMs, BAP	→
					MPO	→
					IL-6, IL-17a	→
	Kawamura et al. [22]	9 UT	20 min Immediately and 1–6 d after Ex	Downhill running (56% VO_2_ max)	Total leukocytes	→
					Neutrophils	→
					Lymphocytes	→
					LmCL	→
					Migratory neutrophils	→

H_2_-saline	Yamazaki et al. [23]	13 horses	2 L 2 h before Ex	High-intensity simulation race	8-OHdG	↓
					d-ROMs, BAP	→
					CK, AST, LDH	→
					Lactate, uric acid	→

H_2_ gas	Nogueira et al. [24]	60 rats	2%·2.4 L/min before and during Ex	Running (80% Vmax)	TNF-*α*, IL-6	↓
					SOD	↑
					TBARS	↓
					NOx	→
					p-CREB	↓

H_2_ water: oral intake of H_2_ water; H_2_-saline: intravenous infusion of H_2_-saline; H_2_ gas: inhalation of H_2_ gas; T: trained; UT: untrained; Ex: exercise; d-ROMs: diacron reactive oxygen metabolites; BAP: biological antioxidant potential; CK: creatine kinase; TBARS: thiobarbituric acid reactive substance; PC: protein carbonyl; TAC: total antioxidant capacity; FFA: free fatty acid; TG: triglyceride; DOMS: delayed-onset muscle soreness; Mb: myoglobin; MPO: myeloperoxidase; IL: interleukin; LmCL: luminol-dependent chemiluminescence; 8-OHdG: 8-hydroxydeoxyguanosine; AST: aspartate aminotransferase; LDH: lactate dehydrogenase; TNF-*α*: tumor necrosis factor-*α*; SOD: superoxide dismutase; NOx: nitrite/nitrate; p-CREB: phosphorylation of cAMP-responsive element binding protein; ↑: increase; →: no change; ↓: decrease.
